# Concurrent Papular-Purpuric Gloves-and-Socks Syndrome and Petechial Bathing-Trunk Eruption Associated With Parvovirus B19 Infection

**DOI:** 10.7759/cureus.112145

**Published:** 2026-07-06

**Authors:** Meryem El Moustaoui, Meryeme Marhraoui, Hajar EL Hassani Taib, Benzekri Laila, Nadia Ismaili

**Affiliations:** 1 Dermatology, Ibn Sina University Hospital Center, Mohammed V University, Rabat, MAR

**Keywords:** dna virus, human parvovirus b19, papular-purpuric gloves and socks syndrome, petechial bathing trunk eruption, symptomatic treatment

## Abstract

Parvovirus B19 infection can manifest with several distinct exanthems, including papular-purpuric gloves-and-socks syndrome (PPGSS). The coexistence of PPGSS and bathing-trunk eruption is uncommon, with very few cases reported in the literature. We describe a previously healthy 23-year-old man who presented with fever, fatigue, and a pruritic eruption. The rash initially involved the pubic area, hands, and feet before spreading to the trunk. Physical examination revealed confluent petechial maculopapular lesions forming a characteristic "gloves-and-socks" distribution, together with a sharply demarcated bathing trunk distribution involving the groin, hips, and buttocks, consistent with a bathing trunk eruption. Laboratory studies showed mild lymphopenia and elevated C-reactive protein, and detection of parvovirus B19 DNA by serum PCR, confirming the diagnosis. The patient received symptomatic treatment with paracetamol, topical antiseptics, and emollients, resulting in complete resolution within 10 days. This case highlights the importance of recognizing these uncommon but characteristic cutaneous manifestations of parvovirus B19 infection to improve diagnostic accuracy and avoid unnecessary investigations and interventions.

## Introduction

Parvovirus B19 infection is associated with a variety of cutaneous manifestations, among which papular-purpuric gloves-and-socks syndrome (PPGSS) is one of the most characteristic [1]. Petechial bathing trunk eruption has also been described in association with parvovirus B19 infection [2]. Their simultaneous occurrence has rarely been reported in the literature. Here, we describe a case of concurrent PPGSS and petechial bathing trunk eruption in an immunocompetent adult.

## Case presentation

A previously healthy 23-year-old man presented with a three‑day history of mild fever (38.2°C), fatigue, and a pruritic skin eruption. The lesions initially appeared on the pubic area, hands, and feet, then gradually spread to the trunk. He received paracetamol at a local emergency department, but the rash progressed, leading to his admission to the dermatology ward. Clinical examination revealed multiple pustules and erythematous‑purpuric patches on the glans penis, scrotum, and perianal region, as well as a moderately pruritic, confluent maculopapular and petechial eruption on the palms (Figure 1) and dorsal surfaces of the hands (Figure 2) and feet (Figure 3) extending beyond the wrists and ankles, forming a classic “gloves‑and‑socks” pattern. Additional petechial macules were noted on the groin (Figure 4), hips, and buttocks, creating a sharply demarcated bathing‑trunk distribution.

**Figure 1:**
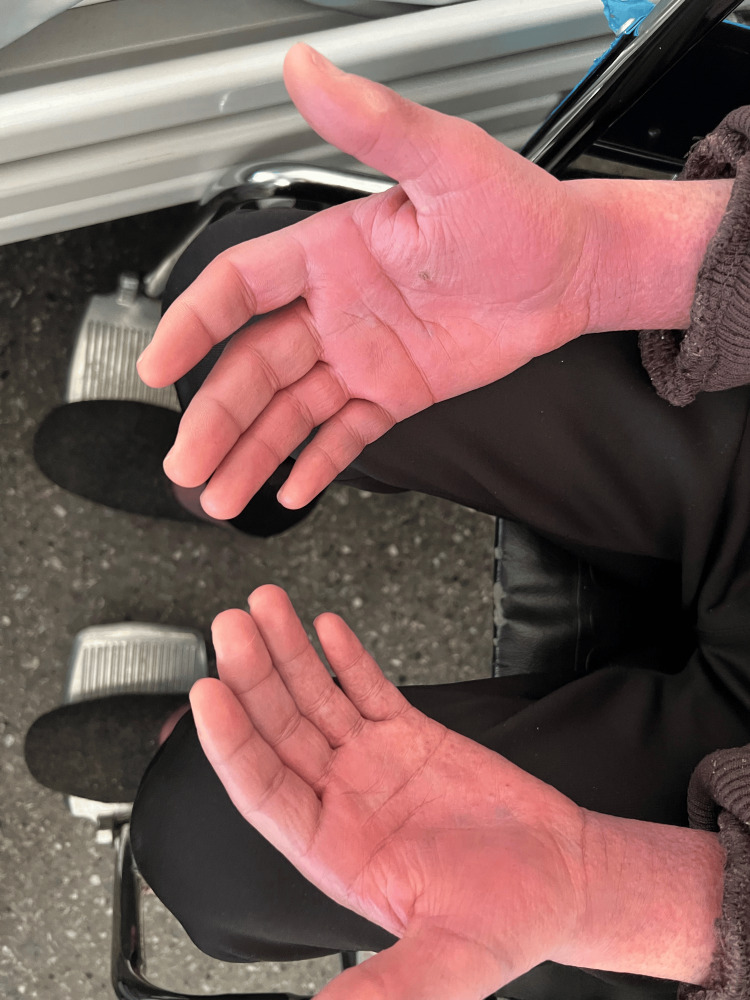
Palmar erythema with edema and petechial purpura forming part of the PPGSS distribution PPGSS: papular-purpuric gloves-and-socks syndrome

**Figure 2:**
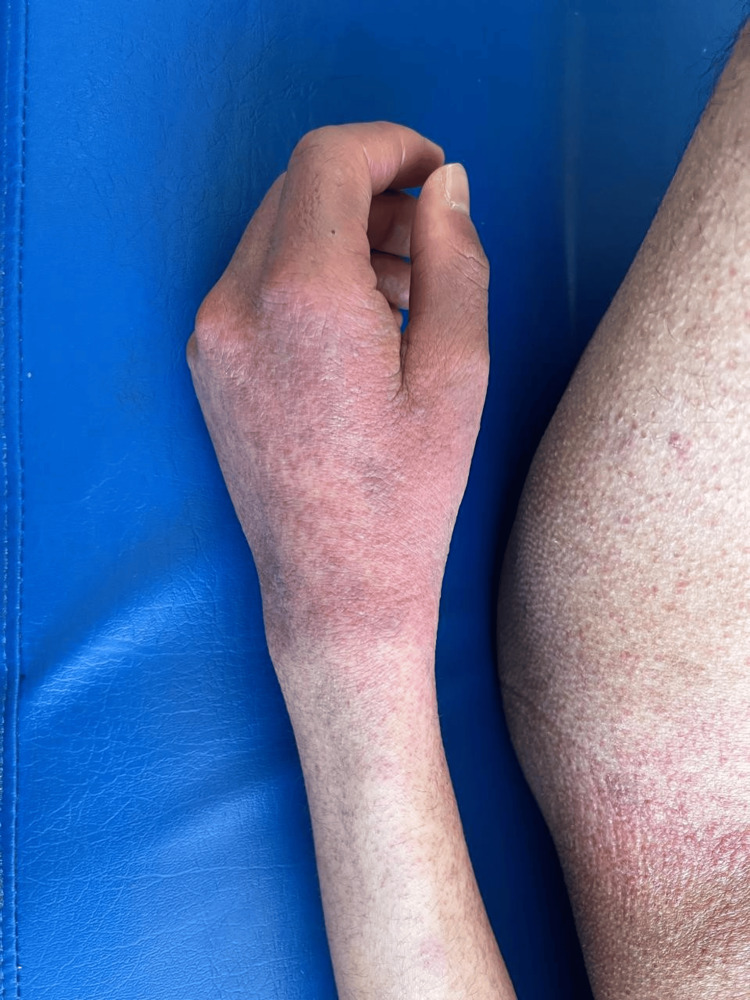
Dorsal involvement of the hands showing erythematous-purpuric lesions with sharp demarcation at the wrists illustrating the typical “gloves” distribution of PPGSS PPGSS: papular-purpuric gloves-and-socks syndrome

**Figure 3:**
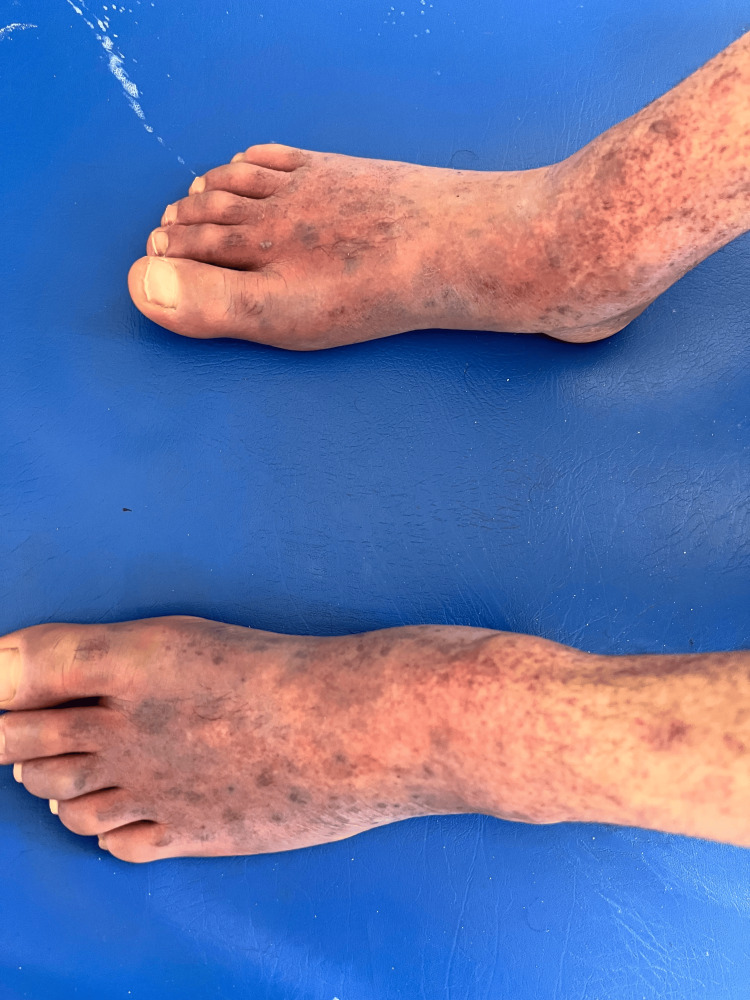
Dorsal involvement of the feet showing erythematous-purpuric lesions

**Figure 4:**
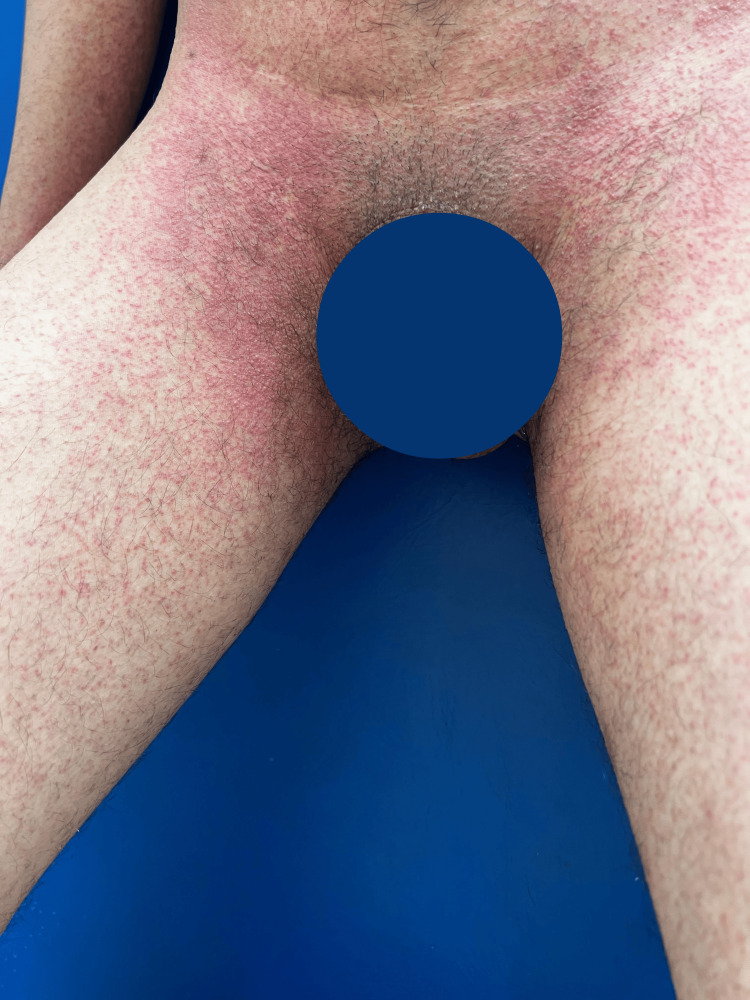
Purpuric eruption involving the pubic region in a bathing-trunk distribution

Laboratory investigations showed mild lymphopenia (700/mm³; reference range: 1500-4000/mm³), markedly elevated erythrocyte sedimentation rate (50 mm/h; reference range: 0-20 mm/h), and elevated C-reactive protein levels (187 mg/L; normal <10 mg/L). Liver and renal function tests were normal.

Serologic tests for hepatitis B and C viruses, human immunodeficiency virus, cytomegalovirus, Epstein-Barr virus, human herpesviruses 6 and 7, and herpes simplex virus were negative. Because parvovirus B19-specific serology was unavailable, recent primary infection could not be confirmed serologically. However, the characteristic clinical presentation together with detectable parvovirus B19 DNA in serum strongly supported the diagnosis of acute parvovirus B19 infection.

A skin biopsy was not performed because the characteristic clinical presentation and serum PCR positive for parvovirus B19 DNA supported the diagnosis. The patient was treated symptomatically with topical antiseptics and paracetamol, and the rash resolved within 10 days.

## Discussion

Parvovirus B19 is a small, non-enveloped, single-stranded DNA virus with tropism for erythroid progenitor cells. It is widespread globally, with greater prevalence in temperate climates [1,3-5]. Infection is transmitted predominantly via respiratory droplets and mainly affects children and young adults, particularly during winter and spring [4,5]. The virus is responsible for several exanthems, including erythema infectiosum and atypical papular-purpuric eruptions [4].

Among these manifestations, PPGSS was first described in 1990 and was subsequently associated with parvovirus B19 one year later; it is characterized by symmetrical erythema and edema of the hands and feet with sharply demarcated purpura [1,3]. The eruption may extend beyond the wrists and ankles and can be accompanied by mucosal involvement such as enanthem, petechiae, erosions, or aphthae. Pruritus, burning sensation, and tingling are frequently reported. Although uncommon, genital involvement has also been described [1,3].

A rarer presentation is the petechial bathing-trunk eruption, characterized by petechial and purpuric macules involving the groin, proximal thighs, buttocks, and lower trunk. The term “bathing trunk” refers to this sharply delimited distribution. This pattern may occur concurrently with PPGSS, as observed in our patient, possibly reflecting a more diffuse immune-mediated vascular reaction induced by the virus [6].

Histopathological findings are generally nonspecific and include perivascular lymphocytic infiltrates, erythrocyte extravasation, occasional parakeratosis, and vacuolar alteration of basal keratinocytes without evidence of true vasculitis [7]. Detection of parvovirus B19 DNA in epidermal or glandular epithelial cells of skin lesions further supports direct viral involvement in the pathogenesis [4,7]. Differential diagnoses include Gianotti-Crosti syndrome, erythema multiforme, hand-foot-and-mouth disease, Henoch-Schönlein purpura, Kawasaki disease, meningococcemia, and drug-induced purpura [6,8].

Although parvovirus B19 infection may lead to systemic complications such as transient aplastic crisis, hepatitis, arthritis, or cardiomyopathy, especially in pregnant or immunocompromised patients [4,5], cutaneous forms such as PPGSS and bathing-trunk eruption usually resolve spontaneously with symptomatic treatment [8].

To our knowledge, the coexistence of PPGSS and petechial bathing-trunk eruption associated with parvovirus B19 infection appears to be rarely reported in the literature (Table 1).

**Table 1:** Comparison of the clinical, laboratory, diagnostic, and therapeutic characteristics of the two previously reported cases of concurrent PPGSS and bathing‑trunk eruption and the present case PPGSS: papular-purpuric gloves-and-socks syndrome, IgM: immunoglobulin M, IgG: immunoglobulin G, PCR: polymerase chain reaction

References	Tidman and Fatima, 2020 [2]	Vázquez-Osorio et al., 2017 [9]	Present case, 2024
Patient sex	Woman	Woman	Man
Patient age	32-year-old	48-year-old	23-year-old
Clinical presentation	Concurrent PPGSS + bathing‑trunk eruption	Concurrent PPGSS + bathing‑trunk eruption	PPGSS + bathing‑trunk eruption
Associated symptoms	Fever, sore throat, arthralgia	Fever, oral lesions	Fever, asthenia
Laboratory findings	Lymphopenia, elevated C-reactive protein, elevated erythrocyte sedimentation rate	Lymphopenia, marked elevation of erythrocyte sedimentation rate and C‐reactive protein, and mildly increased liver enzyme levels	Lymphopenia, elevated C-reactive protein
Diagnosis confirmed by	Parvovirus B19 seroconversion (IgM → IgG)	Parvovirus B19 seroconversion (IgM → IgG)	Serum PCR positive for parvovirus B19 DNA
Treatment	Not mentioned	Topical antiseptics and corticosteroids	Topical antiseptics and paracetamol
Outcome	Complete recovery	Complete resolution within 2 weeks	Complete resolution within 10 days

Tidman and Fatima described an adult woman presenting with symmetric acral and inguinal erythema that progressed to purpura, accompanied by lymphopenia, elevated inflammatory markers, and serological evidence of recent parvovirus B19 infection [2]. Similarly, Vázquez-Osorio et al. reported a 48-year-old woman with concurrent PPGSS and a sharply demarcated bathing-trunk eruption accompanied by mucosal involvement and systemic inflammation [9]. In our patient, the diagnosis was supported by the characteristic clinical presentation and detection of parvovirus B19 DNA by serum PCR. However, parvovirus B19-specific IgM and IgG serology was not performed, which represents a limitation of our report, as serologic confirmation of a recent primary infection was not possible. In addition, a skin biopsy was not performed; although histopathologic findings in PPGSS are generally nonspecific, histopathologic examination could have provided additional supportive evidence.

Nevertheless, the patient's acute febrile illness, typical cutaneous manifestations, and serum PCR positive for parvovirus B19 DNA strongly supported the diagnosis of acute parvovirus B19 infection. Our observation therefore represents an additional case within this very limited clinical spectrum. Altogether, these reports suggest that PPGSS and bathing-trunk eruption likely constitute related manifestations along a shared continuum of parvovirus B19-induced vascular reactivity rather than two separate clinical entities.

## Conclusions

Parvovirus B19 infection may present with concurrent cutaneous manifestations, such as PPGSS and petechial bathing-trunk eruption, in healthy adults. These combined manifestations are generally benign and self-limited. Detection of parvovirus B19 DNA by serum PCR may support the diagnosis when serologic testing is unavailable, although the absence of serology precludes confirmation of a recent primary infection. Recognition of these characteristic cutaneous distributions should prompt consideration of parvovirus B19 infection and facilitate appropriate diagnostic testing while avoiding unnecessary investigations.
